# Efficient Synthesis of 2-Aminopyridine Derivatives: Antibacterial Activity Assessment and Molecular Docking Studies

**DOI:** 10.3390/molecules27113439

**Published:** 2022-05-26

**Authors:** Zahira Kibou, Nadia Aissaoui, Ismail Daoud, Julio A. Seijas, María Pilar Vázquez-Tato, Nihel Klouche Khelil, Noureddine Choukchou-Braham

**Affiliations:** 1 Laboratoire de Catalyse et Synthèse en Chimie Organique, Faculté des Sciences, Université de Tlemcen, BP 119, Tlemcen 13000, Algeria; nbchoukchou@gmail.com; 2 Faculté des Sciences et de la Technologie, Université de Ain Témouchent, BP 284, Ain Témouchent 46000, Algeria; 3 Laboratory of the Sustainable Management of Natural Resources in Arid and Semi Aridareas, University Center Salhi Ahmed Naama, BP 66, Naama 45000, Algeria; aissaoui.nadia@cuniv-naama.dz; 4 Department of Biology, Faculty of Nature and Life, Earth and Universe Sciences, University of Tlemcen, Tlemcen 13000, Algeria; 5 Département des Sciences de la Matière, Université de Mohamed Khider, BP 145 RP, Biskra 07000, Algerie; i.daoud@univ-biskra.dz; 6 Laboratory of Natural and Bio-Active Substances, Faculty of Sciences, University of Tlemcen, BP 119, Tlemcen 13000, Algeria; 7 Departamento de Química Orgánica, Facultad de Ciencias, Universidad de Santiago de Compostela, A da, Alfonso X El Sabio s/n, 27002 Lugo, Spain; pilar.vazquez.tato@usc.es; 8 Laboratory of Applied Microbiology in Food, Biomedical and Environment (LAMAABE), Department of Biology, Faculty of Nature and Life, Earth and Universe Sciences, University of Tlemcen, Tlemcen 13000, Algeria; nklouche2000@gmail.com; 9 Laboratory of Experimental Surgery, Medical Faculty, University of Tlemcen, Tlemcen 13000, Algeria

**Keywords:** 2-aminopyridine derivatives, multicomponent reactions, enaminones, antimicrobial study, molecular docking, ADME-T prediction

## Abstract

A new and suitable multicomponent one-pot reaction was developed for the synthesis of 2-amino-3-cyanopyridine derivatives. Background: This synthesis was demonstrated by the efficient and easy access to a variety of substituted 2-aminopyridines using enaminones as key precursors under solvent-free conditions. Methods: A range of spectroscopic techniques was used to determine and confirm the chemical structures (FTIR, ^1^H NMR, ^13^C NMR). The antimicrobial potency of synthesized compounds (**2a**–**d**) was tested using disk diffusion assays, and the Minimum Inhibitory Concentration (MIC) for the active compounds was determined against a panel of microorganisms, including Gram-positive and Gram-negative bacteria and yeasts. Moreover, a docking analysis was conducted by Molecular Operating Environment (MOE) software to provide supplementary information about the potential, as well as an ADME-T prediction to describe the pharmacokinetic properties of the best compound and its toxicity. Results: The results of the antimicrobial activity indicated that compound **2c** showed the highest activity against Gram-positive bacteria, particularly *S. aureus* and *B. subtilis* whose MIC values were 0.039 ± 0.000 µg·mL^−1^. The results of the theoretical study of compound **2c** were in line with the experimental data and exhibited excellent antibacterial potential. Conclusions: On the basis of the obtained results, compound **2c** can be used as an antibacterial agent model with high antibacterial potency.

## 1. Introduction

**Figure 1:**
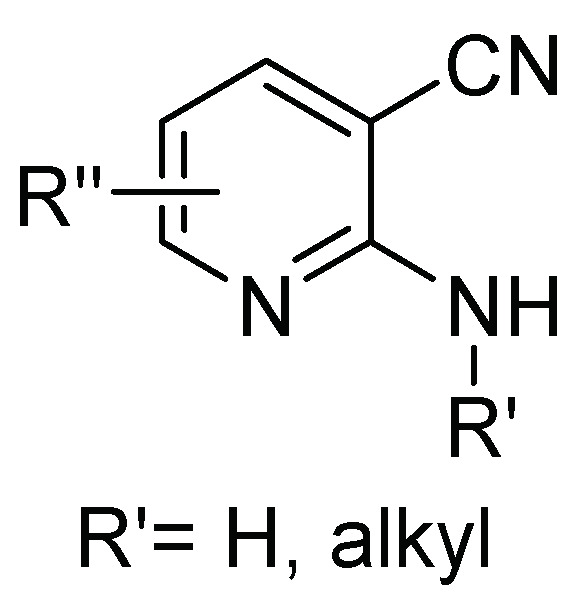
The general structure of 2-amino-3-cyanopyridines.

**Figure 2:**
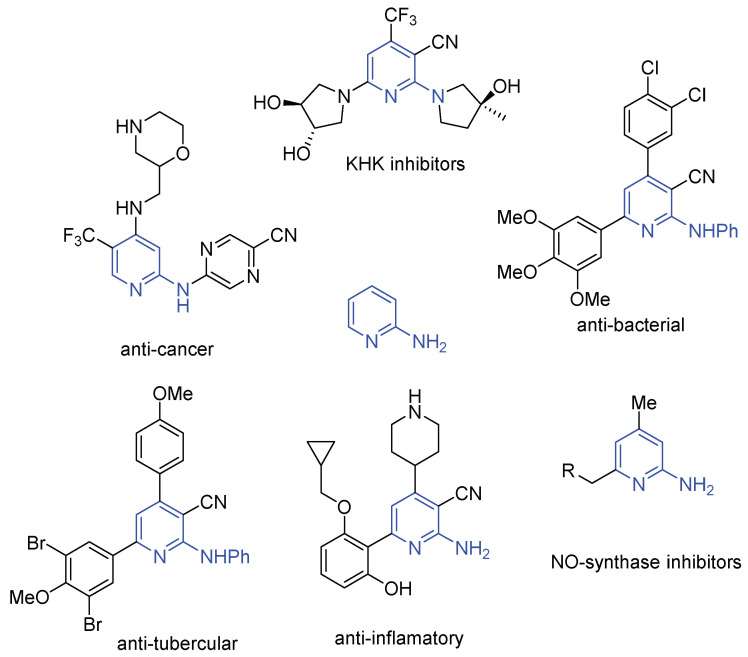
Some bioactive molecules containing the 2-aminopyridine.

Several pyridine compounds are well known and present in many medicinal preparations with various biological profiles, to name only the 2-aminopyridine derivatives (Figure 1) considered precursors for the synthesis of a variety of heterocyclic compounds [1]. Among them, 2-aminopyridines derivatives (Figure 1) have been considered precursors of the synthesis of a variety of heterocyclic compounds [1] and represent effective agents for their antibacterial [2] anticancer [3], anti-inflammatory [4], Ketohexokinase (KHK) inhibitory [5], and NO synthases inhibitory activities [6] (Figure 2). Nowadays, microbial resistance represents one of the most world’s intricate problems due to the increasing rate of mortality and morbidity [7], hence making the synthesis of new compounds to fight multidrug microorganisms with broad activity extremely challenging.

As a part of our interest in the synthesis, we reported a number of clean approaches to prepare building blocks made of 2-aminopyridines [8], 2-pyridones [9], chromenopyridines [10], and pyrimidopyridines [11] from different enamines. Based on these data, multicomponent reactions (MCRs) in a one-pot process give a single product which represents a powerful tool in modern medicinal chemistry. This unique strategy leads to the formation of many new bioactive compounds due to their convergence, rapid process, minimum waste production, and high yields [12].

Recently, many methods for the synthesis of 2-aminopyridines have been developed. However, the MCRs used for the preparation of substituted 2-aminopyridines are very limited. In the continuation of previous work regarding the field of aminopyridine derivates, the present study aims to synthesize new MCRs using enaminones with a simple process of exploring their antimicrobial profile in vitro and computational studies.

## 2. Results and Discussion

This section is divided into subheadings to describe briefly and precisely the experimental results and their interpretation, as well as draw experimental conclusions.

### 2.1. Synthetic Procedures

**Scheme 1:**
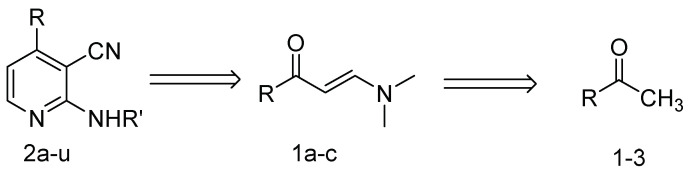
Retrosynthetic scheme of 2-amino-3-cyanopyridines preparation.

Our research group developed new synthetic methodologies for the synthesis of both building blocks and heterocyclic compounds under free solvent conditions. In the previous paper, we reported an efficient method to obtain enaminones as key building blocks from the condensation of methyl ketones with dimethylformamide dimethyl acetal (DMFDMA). Thus, in continuation of our interest in the development of new methodologies, in this article, we describe the synthesis of 3-cyano-aminopyridines in two steps. The synthesis of the target compounds was carried out as illustrated in Scheme 1.

The key substrate enaminones **1a**–**c** are also very versatile intermediates for the synthesis of many heterocycles.The products **1a**–**c** were prepared according to our previous works from acetophenone and an equimolar amount of dimethyl acetal dimethyl-formamide (DMFDMA) under microwave (MW) irradiation and without solvent to obtain **1a**–**c** with excellent yields (80–86%) (Table 1).

The experimental conditions used for the synthesis of 2-amino-3-cyanopyridines were optimized by using enaminone 1, malononitrile, and benzylamine (Table 2, entry 1). Initially, the reactants were introduced without any solvent, and the reaction was allowed to proceed at room temperature for 24 h. However, no products were isolated under these conditions (entry 1). At 40 °C and for 24 h, the reaction provided product **2b** with a yieldof 20% (entry 2). When the temperature was evaluated at 60 °C, the yield of **2b** was increased to 40% (entry 3) for 6 h. The best yield was obtained at 80 °C for 3 h. The structure of **2b** was identified by spectroscopic analysis.

From the optimized reaction, the generality of this MCR was explored using different enaminones **1a**–**c** and primary amines (Table 3) to provide the corresponding products **2a**–**l**. It was found that the synthesis under solvent-free 2-aminopyridine derivatives can be obtained by a simple, fast, and cleaner method using the three-component reaction. Given the exceptional biological properties of heterocyclic aminopyridines, a wide variety of primary amine and enaminones were evaluated, which provided a convenient and flexible method for the synthesis of 2-aminopyridines.

**Scheme 2:**
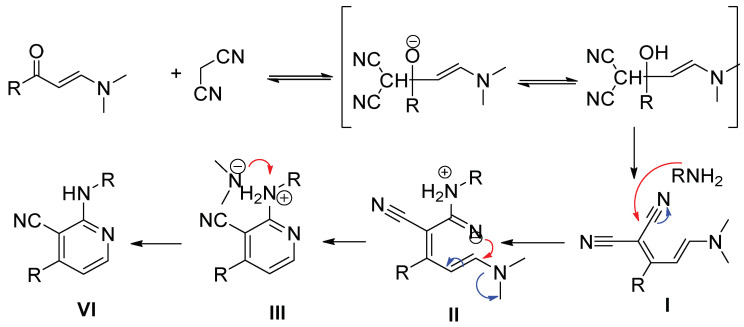
The Proposed Mechanism for the formation of 2-aminopyridines.

The proposed mechanism showed that the enaminone reacts at first with malononitrile via Knoevenagel reaction to afford intermediate I. However, the intermediate I reacted with primary amines at the nitrile groups of intermediate II then the product inter-cyclized to give intermediate III. The reaction is finished with an aromatization step to afford the 2-aminopyridine structure VI (Scheme 2).

The structureof **1a** was confirmed by NH characteristic absorption bands in the IR spectrum of the starting enaminones **1a**–**c**, and the ^1H^ NMR spectra of the same derivatives showed two singlet signals corresponding to NCH_3_ protons at δ 2.94 and 3.01 ppm. The formation of compounds **2a**–**l** was confirmed by spectral data and elemental analyses. The ^1H^ NMR spectra of these derivatives demonstrated the appearance of a new doublet signal at 6.69 ppm corresponding to the CH=CH protons. Mass spectra of these compounds showed distinctive molecular ion peaks at the right m/z values. The IR spectra of these compounds showed the characteristic NH stretching bands in the range of 3361–3365 cm^−1^. In addition, the 1H NMR spectra of the same derivatives showed singlet signals corresponding to NH protons at δ 5.11–5.11 ppm.

### 2.2. Antimicrobial Assay

The antimicrobial activity of each chemical compound was initially tested against twelve target microorganisms. A disk diffusion data assay was employed for primary screening. As shown in Table 4, the highest antimicrobial effect was observed against Gram-positive bacteria, particularly against rod bacteria, including *Bacillus subtilis* ATCC6633, *Listeria monocytogenes* ATCC 15313, and *Bacillus cereus* ATCC 10876. The inhibition zone diameters ranged between 11.33 ± 0.57 mm and 13 ± 0 mm. Compared with the Gentamicin inhibition zone diameter, *Listeria monocytogenes* ATCC 15313 was found topossess a similar diameter using a Gentamicin half-concentration. Moderate activity against target cocci Gram-positive bacteria was observed, and the diameters of the inhibition zones were between 8.66 ± 0.57 mm to 9.66 ± 0. However, no effect was observed against Gram-negative bacteria and two yeasts. These results were obtained from compound **2c**, while no activity was detected from **2a**, **2b**, and **2d**, which represented the compound **2c** analogs. These results can suggest the following: (i) antimicrobial activity is due to cyclohexylamine presence, and (ii) in a series of 2-aminopyridine compounds, the introduction of other CH_2_ after amine function was responsible for the activity disappearance.

Compound **2c**’s selective activity against only Gram-positive bacteria can occur due to the cell’s structure. It is known that Gram-positive bacteria are more sensitive than Gram-negative ones [13] because the cell structure of their wall, with a principal share of peptidoglycan, p enables the hydrophobic compounds to infiltrate the cells and proceed on the wall as well as on the cell membrane and inside the cytoplasm. The cell structure of the Gram-negative bacteria’swall is more complex with a reduced amount of peptidoglycan and with an external membrane composed of a phospholipids’ double-layer connected with the internal membrane by lip polysaccharides [14]. Therefore, Gram-negative bacteria present a further complex biological barrier that avoids the penetration of different antibiotics, whereas their periplasmic space has proteins and enzymes that are able to break down foreign molecules [15].

The disk diffusion assay is the primary screening representing a rapid and qualitative method to detect the antimicrobial potency of compounds. For a quantitative assessment, the MIC assay was done using the active chemical compound **2c**, and the results are listed in Table 5. Overall, the most Gram-positive bacteria-sensitive strains were *S. aureus* and *B. subtilis,* with MIC values of 39 ± 0.000 µg·mL^−1^. *B.cereus*, *E. faecalis*, and *M. luteus* expressed sensitivity with MIC values of 78 ± 0.000 µg·mL^−1^. However, *L. monocytogenes* exhibited a moderate sensitivity with a MIC value of156 ± 0.000 µg·mL^−1^ in comparison to the MIC values of the standard drug Gentamicin.

These data are in agreement with Chikhalia’s and Patel’s results [16], which indicated the presence of cyclohexylamine generated antibacterial activity towards Gram-positive bacteria. However, MICs values were significantly higher compared to those of the present study.

The mechanism responsible for the selective antibacterial profile of the **2c** compound is unknown at the moment, and then we speculated that the antibacterial potency might have been related to the global charge of the compound, in particular to the positive charge of carbon directly connected to the cyclic compound. The cationic head groups bind and disrupt the bacterial cell membrane with the aid of hydrophobic and electrostatic interactions leading to the release of cytoplasmic constituents and the death of the cell [17]. Work is in progress to provide a detailed clarification as to theantibacterial action mechanism of the effective compound.

### 2.3. Molecular Docking Studies

The detailed results of compound **2c**’s docking simulation with both X-ray crystals of *S. aureus* (PDB ID: 4URM) and *B. subtilis* (PDB ID: 2RHL) targets aresummarized in Table 6. To estimate all possible interactions, the docking outputs generated by MOE software were converted into (.pdb) files and visualized with the default parameters of the BIOVIA DS visualizer package (Dassault Systèmes BIOVIA, Discovery Studio Modeling Environment, 2020).

In general, compound **2c**’sdocking results demonstrated a good interaction with two target proteins, the *S. aureus* (PDB ID: 4URM) and *B. subtilis* (PDB ID: 2RHL) targets. This confirms that compound **2c** fits well in the binding pockets of these bacterial targets.

According to the docking results (Table 7), compound **2c** is identified to bind to the *S. aureus* ATP binding pocket with a score value very close to native KBD (kibdelomycin) (−5.532 vs. −6.383 kcal/mol)). In addition, it was noted that this compound established the interactions with *B. subtilis* pocket with a score value very close to native GDP (kibdelomycin) (−6.389 vs. −7.843 kcal/mol)).

**Figure 3:**
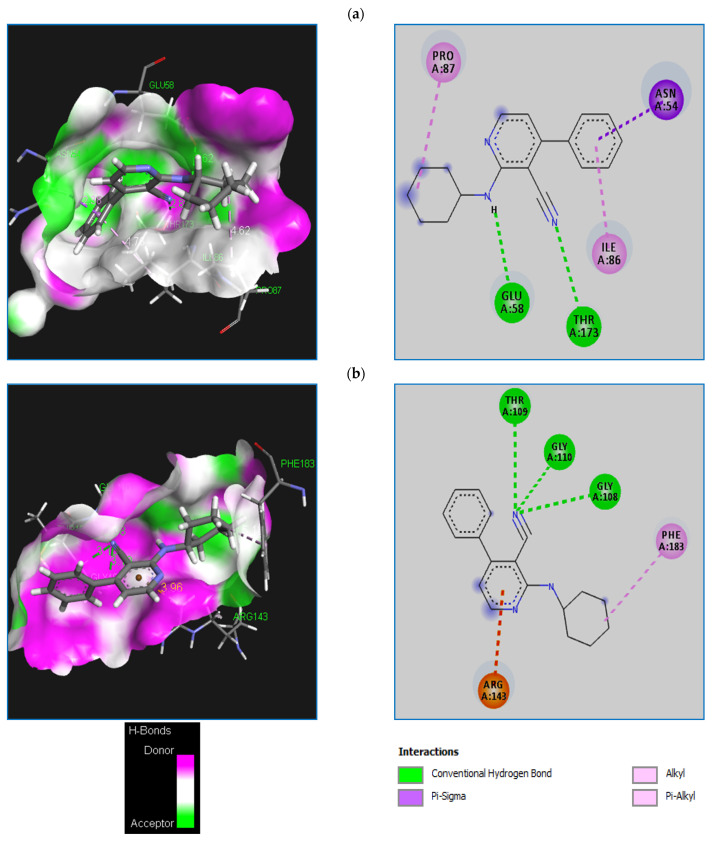
2D and 3D Binding Pose and the Interaction of Compound **2c** with: (**a**) the active site residues of *S. aureus* (PDB ID: 4URM), (**b**) the active site residues of *B. subtilis* (PDB ID: 2RHL).

As presented in Figure 3, compound **2c** binds to the same site as KBD and GDP, but with different residues, respectively. It is stabilized by several hydrogen-bonding interactions and close hydrophobic contacts with surrounding amino acids.

Based on the antimicrobial assay results (Table 3 and Table 4), compound **2c** represents the activity against *S. aureus* and *B. subtilis* and established several interactions with the active site residues of *S. Aureus* (PDB ID: 4URM) and *B. subtilis* (PDB ID: 2RHL) targets.

The compound **2c**’s docking conformation showed that itestablished five interactions with *S. aureus* active site residues (PDB ID: 4URM). Two strong conventional H-bonds [18,19] were observed: the first one was observed between the hydrogen atom of the compound and active site residues GLU(A:58) with a bond distance of 2.62 Å, and the secondwas established between the oxygen atom of the compound and the active site residues THR(A:173) with a bond distance of 2.88 Å. We can clearly see these two residues: GLU(A:58) and THR(A:173), play an important role in the active site of *S. aureus* (PDB ID: 4URM), which have been reported inprevious studies [20,21].

A Pi–Sigma interaction formed with active site residues; ASN(A:54) has a bond distanceequal to 2.38 Å (Figure 3). Two other weak interactions of hydrophobic types appeared: the first is of the type Pi–alkyl and was established with six rings of the compound and active site residues ILE(A:86) with a bond distance of 4.76 Å, and the second, which is of the type Alkyl formed the carbon atom of the compound and active site residues PRO(A:87) with abond distance of 4.62 Å (Table 7 and Figure 3). Furthermore, Rahman, M. et al. [22] and Pham, E.C. et al. [23] confirmed that ASN(A:54), PRO(A:87) and ILE(A:86) are responsible forthe formation of different interactions in the binding site of the target.

In addition, compound **2c**’s docking results showed good interactions with the second target (*B. subtilis*), especially because it established five interactions with *B. subtilis* active site residues (PDB ID: 2RHL).Three strong conventional H-bonds [18,19] were observed between the nitrogen atom of the compound and three active site residues GLY(A:110), GLY(A:108), and THR(A:109) with bond distances of 2.38 Å, 3.10 Å, and 3.10 Å, respectively. We note that these results were confirmed by Singh, D. et al. [24]. Pi–cation interactions formed between six rings of the compound with active site residues ARG(A:143) with a bond distance of 3.96 Å (Figure 3). Pi–alkyl type is another weak interaction formed between the carbon atom of the compound and active site residues PHE(A:183) with a bond distance of 5.18 Å (Table 7 and Figure 3). In addition, Matsui, T. et al. [25] confirmed that ARG(A:143) and PHE(A:183) are the keysto the majority of interactions. Finally, fora comparison, compound **2c**’s binding mode in the pocket sites of the *S. aureus* (PDB ID: 4URM) and *B. subtilis* (PDB ID: 2RHL) targets, and in line with our expectations, compound **2c** is predicted to bind to the active site residues of both bacterial targets with high affinities compared to the native compounds.

According to the docking studies, there was sufficient evidence that compound **2c** forms several H-bond interactions with *S. aureus* (PDB ID: 4URM) and *B. subtilis* (PDB ID: 2RHL) targets and should exhibit the inhibition of both bacterial targets. However, this compound showed strong inhibitory activities when tested against Gram-positive bacteria, particularly against *S. aureus* and *B. subtilis,* ofwhich the MIC values were 0.039 ± 0.000 µg·mL^−1^. This might be one of the reasons this compound showed biological activity and different interactions with both target proteins.

### 2.4. ADMET and Drug-Likeness Prediction

Drug-likeness and physicochemical properties of compound **2c** were evaluated by checking Lipinski’s rule of five and the Veber and Ghose rules, which are crucial for rational drug design. In addition, absorption, distribution, metabolism, excretion, and toxicity (ADMET) are crucial parameters for the drug development process. All results are given in Table 7.

It is apparent from this table that compound 2c has a number of hydrogen bond donors < 7 (n-HD: (0~7)) and hydrogen bond acceptors < 12 (n-HA: (0~12)).Furthermore, the molecular weight of the compound belongs to the interval: 100~500 g/mol, and the MLogPand WLogP values are <5. Further analysis of the table revealed that the compound 2c exhibited three rotational bonds (nROTB) (should be <10) and a topological polar area value (TPSA) of 48.71 Å (less than 140 Å), justifying the flexibility of the molecule and its good permeability in the cellular plasma membrane to cross the blood−brain barrier (BBB).

In addition, this result indicates that compound **2c** satisfies all of the criteria of drug-likeness without any violation of the Lipinski, Veber, and Egan rules.

According to the literature [26,27], the process of drug development must use suitable parameters that represent the ADMET properties. An effective candidate drug may sometimes be effective against the therapeutic target (affinity and selectivity), but this is not sufficient—it must be safe and have adequate ADME qualities in a therapeutic concentration.

As can be seen from the above table, compound **2c** has an average Caco-2 permeability, and HIA value is higher than 30%, suggesting that the compound can be administered orally and is strongly absorbed by the gastrointestinal system into the bloodstream. We also note that the logPS value of the compound is between −2 < logPS < 0, which means that compound **2c** is able to penetrate the CNS. Additionally, the logBB value of this compound is 0.22; this indicates that the compound presents an average distribution in the brain (Table 7).

The inhibition of one of the cytochrome P450 (CYP) isoforms may alter drug metabolism, while a lack of inhibition may mean that the compounds will not alter the metabolism of other substances [28]. Compound **2c** will interact with some Cytochrome P450 isoforms (CYP1A2 and CYP2D6) with the exception of CYP2C19. In addition, this compound is not likely to obstruct the organic cation transporter substrate (OCT2), and it has a low excretion time. Finally, the compound is not likely to be cardiotoxic as it does not obstruct the hERG K+ channels linked to deadly cardiac arrhythmias [29], and no AMES toxicity was found (Table 7).

## 3. Materials and Methods

All of the products were prepared in our laboratory and analyzed by spectroscopic methods. The melting points were measured using a Bank KoflerHeizbank apparatus standard WME 50–260 °C without particular correction. IR spectra were performed on solid samples using a Fourier transform Perkin Elmer Spectrum with an ATR accessory. Only significant absorptions were listed. The ^1^H and ^13^C NMR spectra were recorded on Bruker AC 400 spectrometers at 400 and 100 MHz, respectively. The samples were recorded in CDCl_3_ solutions using TMS as an internal standard. The chemical shifts are expressed in δ units (ppm) and quoted downfield from TMS. The multiplicities are reported as s: singlet, d: doublet, t: triplet, q: quartet, m: multiplet.

### 3.1. General Synthesis of Enaminones ***1a***–***c***

**Procedure 1**: Enaminone synthesis with an equimolar mixture of carbonyl compound **1**–**3** (1 mmol) and (1 mmol) of DMFDMA was irradiated under microwave conditions for 5 min. After cooling, a yellow crystalline precipitate was formed, then filtered off, washed with Et_2_O, and dried to give corresponding enaminones.

- *3-(Dimethylamino)−1-phenylprop-2-en−1-one ***1a****

The general procedure 1 using (1 mmol) of **1** and (1 mmol) of DMFDMA, gave 86% of compound **1a** as yellow solid, mp 90 °C. IR ν_max_ cm^−1^: 1582 (C=C); 1633 (C=O); ^1^H NMR (400 MHz, CDCl_3_): 7.90–7.87 (2H, m), 7.80 (1H, d, *J*_H-H_ = 12.40 Hz), 7.45–7.37 (3H, m), 5.71 (1H, d, *J*_H-H_ = 12.4 Hz), 3.12 (s, 3H), 2.91 (s, 3H). ^13^C NMR (100 MHz, CDCl_3_): 187.72, 153.24, 139.53, 129.85, 127.10, 126.47, 91.24, 44.00, 36.27. IR (neat, cm^−1^): 1633, 1582. EIMS *m*/*z* 175 (M + H, 34), 158 (84), 98 (100). EIMS *m*/*z* (% relative abundance) 176 (M + H, 36), 98 (54),77 (28). (ES-QTOF) Calcd for C_11_H_13_NO M + H 176.1123 Found 176.1117.

- *3-(Dimethylamino)−1-(4-methoxyphenyl)prop-2-en−1-one ***1b****

The general procedure 1 using (1 mmol) of **2** and (1 mmol) of DMFDMA, gave 70% of compound **1b** as yellow solid, mp 98–99 °C. IR ν_max_ cm^−11^: 1541 (C=C); 1648 (C=O); ^1^H NMR (400 MHz, CDCl_3_): 2.91 (3H, s, NCH_3_); 3.01 (3H, s, NCH_3_); 3.85 (3H,s, OCH_3_); 5.65 (1H, d, *J*_H-H_ = 12.40 Hz, CH=CH); 6.85 (1H, d, *J*_H-H_ = 12.40 Hz, CH=CH); 7.78 (2H, d, *J*_H-H_ = 8.81, H_arom_), 7.85 (2H, d, *J*_H-H_ = 8.81 Hz, H_arom_);^13^C NMR (100 MHz, CDCl_3_): 36.32; 44.11; 55.43; 90.23; 126.38; 127.10; 129.74; 139.53; 152.30; 187.23. EIMS *m*/*z* (% relative abundance) 176 (M + H, 98), 77 (28). (ES-QTOF) Calcd for C_12_H_15_NO_2_M + H 206.1125 Found 206.1115.

- *3-(Dimethylamino)−1-p-tolylprop-2-en−1-one ***1c****

The general procedure 1 using (1 mmol) of **3** and (1 mmol) of DMFDMA, gave 80% of compound **1c** as yellow solid, mp 91 °C. IR ν_max_ cm^−1^:1539 (C=C); 1647 (C=O); ^1^H NMR (400 MHz, CDCl_3_): 2.42 (3H, s, Ph–CH_3_); 2.94 (3H, s, NCH_3_); 3.41 (3H, s, NCH_3_); 5.64 (1H, d, *J*_H-H_ = 12.40 Hz, CH=CH); 7.18 (1H, d, *J*_H-H_ = 12.40 Hz, CH=CH); 7.58 (2H, d, *J*_H-H_ = 8.3, H_arom_); 7.75 (2H, d, H_arom_); ^13^C NMR (100 MHz, CDCl_3_): 24.33; 36.27; 44.00; 91.24; 126.47; 127.10; 129.85; 139.53; 153.24; 187.72. EIMS *m*/*z* (% relative abundance) 190 (M + H, 33), 172 (87), 120 (26), 99 (100), (ES-QTOF) Calcd for C_12_H_16_NO M + H 190.1126; Found 190.1122.

### 3.2. General Synthesis of 3-Cyano-2aminopyridones ***2a***–***l***

**Procedure 2**: An equimolar mixture of enaminones **1**–**3** (1 mmol), primary amine (1 mmol), and malononitrile (1 mmol) was heated for 3 h under solvent-freeconditions. After cooling and the completion of the reaction, the residue obtained was washed several times with diethyl ether to give the desired 2-aminpyridines **2a**–**l**.

- *2-Phenethylamino-4-phenyl-nicotinonitrile ***2a****

The general procedure 2 using (1 mmol) of **1**, (1 mmol) of malononitrile and (1 mmol) of phenethylamine, gave 66% of compound **2a** as yellow solid, mp 102 °C. IR ν_max_ cm^−1^:1540 (C=C); 1656 (C=O); 2219 (CN); 3365 (NH); ^1^H NMR (400 MHz, CDCl_3_): 2.98 (2H, m, CH_2_–CH_2_), 2.83 (2H, m, CH_2_=CH_2_); 5.11 (1H, t, *J*_H-H_ = 4.6 Hz, NH–CH_2_); 6.69 (1H, d, *J*_H-H_ = 4.6 Hz, CH=CH); 7.29–7.32 (5H, m, H_arom_); 7.45–7.60 (5H, m, H_arom_); 8.33 (1H, d, *J*_H-H_ = 4.6 Hz, CH=CH); ^13^C NMR (100 MHz, CDCl_3_): 39.2; 42.50; 90.16; 113.14; 116.92; 127.45–128.62; 128.76–129.79; 135.66; 138.80; 153.05; 155.33; 160.25. MS *m/z* (% relative abundance): 300 (M + H, 100), 135 (33), (ES-QTOF) Calcd for C_21_H_17_N_3_M + H 300.1128; Found 300.1125.

- *2-(Benzylamino)-4-phenylpyridine-3-carbonitrile ***2b****

The general procedure 2 using (1 mmol) of **1**, (1 mmol) of malononitrile and (1 mmol) of benzylamine, gave 75% of compound **2b** as white solid, mp 93 °C. IR ν_max_ cm^−1^: 1575 (C=C); 1531 (C=C); 2215 (CN); 3361 (NH); ^1^H NMR (400 MHz, CDCl_3_): 4.76 (2H, d, *J*_H-H_ = 5.2 Hz, NH–CH_2_); 5.60 (1H, t, *J*_H-H_ = 4.6 Hz, NH–CH_2_); 6.69 (1H, d, *J*_H-H_ = 4.6 Hz, CH=CH); 7.28–7.30 (5H, m, H_arom_); 7.46–7.58 (5H, m, H_arom_); 8.30 (1H, d, *J*_H-H_ = 4.6 Hz, CH=CH); ^13^C NMR(CDCl_3_) _C:_ 45.60; 90.16; 113.14; 116.92; 127.56–128.21; 128.76–129.81; 136.78; 138.49; 152.07; 154.50; 159.26. MS *m*/*z* (% relative abundance): 286 (M + H, 100), 208 (23), 91 (93).Calcd for C_19_H_16_N_3_M + H 286.1344; Found 286.1345.

- *2-(cyclohexylamino)-4-phenylnicotinonitrile ***2c****

The general procedure 2 using (1 mmol) of **1**, (1 mmol) of malononitrile and (1 mmol) of cyclohexylamine, gave 80% of compound **2b** yellow solid, mp 188–189 °C; IR ν_max_ cm^−1^:1571 (C=C); 1532 (C=C); 2219 (CN); 3365 (NH); ^1^H NMR(CDCl_3_) 1.49–1.75 (m, 2H, -(CH_2_)_4_-); 1.75 (1H, q, CH-NH); 5.22 (1H, s, NH); 6.33 (1H, d, *J*_H-H_ = 5.8 Hz, CH=CH-N); 7.325–7.56 (5H, m, H_arom_); 8.26 (1H, d, *J*_H-H_= 5.7 Hz, CH=CH-N); ^13^C NMR(CDCl_3_) _C:_ 11,115; 24.56; 46.53; 87.2; 105.13; 116.21; 127.33−129.4; 153.35; 155.39; 156.23; EIMS *m*/*z* (% relative abundance): 277 (M + H, 100), (19), HRMS (ESI-QTOF): Calcd for: C_18_H_19_N_3_M + H 277.1322; Found: 277.1123.

- *2-(Butylamino)-4-phenylnicotinonitrile ***2d****

The general procedure 2 using (1 mmol) of **1**, (1 mmol) of malononitrile and (1 mmol) of butylamine, gave 61% of compound **2b** white solid, mp 159 °CIR ν_max_ cm^−1^: 1583 (C=C), 1554 (C=C); 2219 (CN); 3383 (NH); ^1^H NMR (CDCl_3_): 0.91 (3H, t, *J*_H-H_ = 7,2Hz, -(CH_2_)_3_–CH_3_); 1.32 (2H, m, -N–CH_2_–CH_2_–CH_2_–CH_3_); 1.33 (2H, m, N–CH_2_–CH_2_–CH_2_ –CH_3_); 2.64 (2H, t, *J*_H-H_ = 4.20 Hz N–CH_2_–CH_2_–CH_2_ –CH_3_); 5.36 (1H, t, *J*_H-H_= 4.20 Hz, NH); 6.63 (1H, d, *J*_H-H_ = 5.8 Hz, CH=CH-N); 7.35–7.56 (5H, m, H_arom_); 8.31 (1H, d, *J*_H-H_ = 5.8 Hz, CH=CH-N); ^13^C NMR (CDCl_3_): 13.71; 21.31; 32.62; 41.5; 88.3; 113.42; 117.01; 126.25–129.86; 136.88; 152.08; 155.34; 160.12. EIMS *m/z* (% relative abundance): 252 (M + H, 100), (23), HRMS (ESI-QTOF): Calcd for: C_16_H_18_N_3_M + H 252.1321; Found: 252.1123.

- *4-(4-Methoxy-phenyl)-2-phenethylamino-nicotinonitrile ***2e****

The general procedure 2 using (1 mmol) of **2**, (1 mmol) of malononitrile and (1 mmol) of phenethylamine, gave 70% of compound **2e** as yellow solid, mp 113 °C. IR ν_max_ cm^−1^:1541(C=C); 1654(C=O); 2217 (CN); 3364 (NH); ^1^H NMR (400 MHz, CDCl_3_): 2.16 (3H, s, OCH_3_); 2.98 (2H, m, CH_2_–CH_2_), 2.83 (2H,m, CH_2_=CH_2_); 5.11 (1H, t, *J*_H-H_ = 4.6 Hz, NH–CH_2_); 6.69 (1H, d, *J*_H-H_ = 4.6 Hz, CH=CH); 7.29–7.32 (5H, m, H_arom_); 7.45–7.60 (5H, m, H_arom_); 8.33 (1H, d, *J*_H-H_ = 4.6 Hz, CH=CH); ^13^C NMR (100 MHz, CDCl_3_): 39.2; 42.50; 90.16; 113.14; 116.92; 127.45–128.62; 128.76–129.79; 135.66; 138.80; 153.05; 155.33; 160.25. MS *m/z* (% relative abundance): 330 (M + H, 100), 128 (25), (ES-QTOF) Calcd for C_21_H_20_N_3_M + H 330.1123; Found 300.1121.

- *2-(Benzylamine)-4-(4-methoxyphenyl)nicotinonitrile ***2f****

The general procedure 2 using (1 mmol) of enaminone **2**, (1 mmol) of malononitrile and (1 mmol) of benzylamine, gave 80% of compound **2f** as white solid, mp 189 °C. IR ν_max_ cm^−1^: 1575 (C=C); 1531 (C=C); 2215 (CN); 3361 (NH); ^1^H NMR (400 MHz, CDCl_3_): 2.17 (3H, s, OCH_3_); 4.73 (2H, d, *J*_H-H_ = 5.2 Hz, NH–CH_2_); 5.63 (1H, t, *J*_H-H_ = 5.2 Hz, NH–CH_2_); 6.70 (1H, d, *J*_H-H_ = 6.1 Hz, CH=CH); 7.25–7.32 (5H, m, H_arom_); 7.46–7.58 (4H, m, H_arom_); 8.32 (1H, d, *J*_H-H_ = 6.1 Hz, CH=CH); ^13^C NMR(CDCl_3_): 45.65; 55.60; 91.26; 113.25; 116.33; 127.86–129.23; 129.76–130.12; 136.55; 137.79; 153.15; 155.33; 159.58; MS *m/z* (% relative abundance): 316 (M + H, 100), 158 (25); Calcd for C_20_H_18_N_3_O M + H 316.1450; Found 316.1450.

- *2-Cyclohexylamino-4-(4-methoxy-phenyl)-nicotinonitrile ***2g****

The general procedure 2 using (1 mmol) of **1**, (1 mmol) of malononitrile and (1 mmol) of cyclohexylamine, gave 78% of compound **2g** yellow solid, mp 190 °C; IR ν_max_ cm^−1^ 1569 (C=C); 1535 (C=C); 2217 (CN); 3364 (NH); ^1^H NMR(CDCl_3_): 1.49–1.75 (m, 2H, -(CH_2_)_4_-); 1.75 (1H, q, CH-NH); 2.17 (3H, s, OCH_3_); 5.22 (1H, s, NH); 6.33 (1H, d, *J*_H-H_= 5.8 Hz, CH=CH-N); 7.325–7.56 (5H, m, H_arom_); 8.26 (1H, d, *J*_H-H_ = 5.7 Hz, CH=CH-N); ^13^C NMR (CDCl_3_) _C:_ 11.117; 23.46; 45.53; 87.2; 105.13; 116.21; 127.36−129.40;153.35; 155.39; 156.25; EIMS *m*/*z* (% relative abundance): 308 (M + H, 100), (19), HRMS (ESI-QTOF): Calcd for: C_19_H_22_N_3_M + H 308.1317; Found: 308.1113.

- *2-(Butylamino)-4-(4-methoxyphenyl)nicotinonitrile ***2h****

The general procedure 2 using (1 mmol) of enaminone **2**, (1 mmol) of malononitrile and (1 mmol) of butylamine, gave 72% of compound **2f** as white solid, mp 201 °C. IR ν_max_ cm^−1^: 1560 (C=C); 1545 (C=C); 2218 (CN); 3365 (NH); ^1^H NMR (400 MHz, CDCl_3_): 0.95 (3H, t, *J*_H-H_ = 5.3 Hz, -(CH_2_)_3_ (3H, s, OCH_3_); 1.34 (2H, m, -N–CH_2_–CH_2_–CH_2_–CH_3_); 2.44 (2H, t, *J*_H-H_ = 4.30 Hz N–CH_2_–CH_2_–CH_3_);); 5.63 (1H, t, *J*_H-H_ = 7.2 Hz, -(CH_2_)_3_–CH_3_); 6.70 (1H, d, *J*_H-H_ = 6.1 Hz, CH=CH); 7.25–7.32 (5H, m, H_arom_); 7.43–7.60 (4H, m, H_arom_); 8.32 (1H, d, *J*_H-H_ = 6.1 Hz, CH=CH); ^13^C NMR(CDCl_3_): 13.25; 24.44; 55.60; 91.26; 113.25; 116.33; 127.86–129.23; 129.76–130.12; 136.55; 137.79; 153.15; 155.33; 159.58; MS *m/z* (% relative abundance): 316 (M + H, 100), 158 (25); Calcd for C_17_H_20_N_3_O M + H 282.1550; Found 282.1350.

- *2-Phenethylamino-4-p-tolyl-nicotinonitrile ***2i****

The general procedure 2 using (1 mmol) of **2**, (1 mmol) of malononitrile and (1 mmol) of phenethylamine, gave 73% of compound **2i** as yellow solid, mp 113 °C. IR ν_max_ cm^−1^:1540 (C=C); 1654 (C=O); 2219 (CN); 3365 (NH); ^1^H NMR (400 MHz, CDCl_3_): 2.16 (3H, s, OCH_3_); 2.34 (3H, s, –CH_3_); 2.98 (2H, m, CH_2_–CH_2_), 2.83 (2H, m, CH_2_=CH_2_); 5.11 (1H, t, *J*_H-H_ = 4.6 Hz, NH–CH_2_); 6.70 (1H, d, *J*_H-H_ = 4.6 Hz, CH=CH); 7.30–7.32 (5H, m, H_arom_); 7.44–7.60 (5H, m, H_arom_); 8.31 (1H, d, *J*_H-H_ = 4.6 Hz, CH=CH); ^13^C NMR (100 MHz, CDCl_3_): 39.2; 42.50; 90.16; 113.14; 116.92; 127.45–128.62; 128.76–129.79; 135.66; 138.80; 153.05; 155.33; 160.25. MS *m/z* (% relative abundance): 344 (M + H, 100), 128 (25), (ES-QTOF) Calcd for C_23_H_25_N_3_M + H 344.1123; Found 344.1121.

- *2-(Benzylamine)-4-p-tolylnicotinonitrile ***2j****

The general procedure 2 using (1 mmol) of **1**, (1 mmol) of malononitrile and (1 mmol) of benzylamine, gave 71% of compound **2j** as white solid, mp 189 °C; IR ν_max_ cm^−1^:1578, 1533 (C=C); 2219 (CN); 3365 (NH); RMN ^1^H (CDCl_3_): 2.39 (3H, s, Ph–CH_3_); 4.68 (2H, d, *J*_H-H_ = 4.6 Hz, NH–CH_2_); 5.65 (1H, t, *J*_H-H_ = 4.6 Hz, NH–CH_2_); 6.72 (1H, d, *J*_H-H_ = 4.6 Hz, CH=CH); 7.24–7.32 (5H, m, H_arom_); 7.44–7.56 (4H, m, H_arom_); 8.34 (1H, d, *J*_H-H_ = 4.6 Hz, CH=CH); RMN ^13^C (CDCl_3_): 24.54; 45.65; 91.13; 113.41; 116.87; 127.35–127.98; 128.12–129.53; 135.98; 139.19; 153.17; 154.59; 160.15; EIMS *m*/*z* (% relative abundance): 300 (M + H, 80), 150 (25), HRMS (ESI-QTOF): Calcd for: C_20_H_18_N_3_M + H 300.1243; Found: 300.1344.

- *2-(Cyclohexylamino)-4-p-tolylnicotinonitrile ***2k****

The general procedure 2 using (1 mmol) of **1**, (1 mmol) of malononitrile and (1 mmol) of cyclohexylamine, gave 75% of compound **2k** as yellow solid, mp 178 °C; IR _max_ (neat/cm^−1^): 1574.1531 (C=C); 2219 (CN); 3364 (NH); RMN ^1^H (CDCl_3_) ^1^_H_:1.47–1.75 (m, 2H, -(CH_2_)_4_-); 1.75 (1H, q, CH-NH); 2.34 (3H, s, –CH_3_); 5.22 (1H, s, NH); 6.32 (1H, d, *J*_H-H_= 5.8 Hz, CH=CH-N); 7.325–7.56 (5H, m, H_arom_); 8.26 (1H, d, *J*_H-H_ = 5.7 Hz, CH=CH-N); RMN ^13^C (CDCl_3_) d_C_: 11.12; 23.60; 24.41; 46.51; 88.3; 105.11; 117.01; 127.32−129.3; 152.38; 155.34; 156.22; EIMS *m*/*z* (% relative abundance): 291 (M + H, 100), (19), HRMS (ESI-QTOF): Calcd for: C_19_H_21_N_3_M + H 291.1224; Found: 291.1155.

- *2-Butylamino-4-p-tolyl-nicotinonitrile ***2l****

The general procedure 2 using (1 mmol) of **1**, (1 mmol) of malononitrile and (1 mmol) of butyl amine, gave 61% of compound **2l** as white solid, mp 161 °C; IR ν_max_ cm^−1^: 1583 (C=C), 1554 (C=C); 2217 (CN); 3381 (NH); ^1^H NMR(CDCl_3_): 0.92 (3H, t, *J*_H-H_ = 7.2 Hz,–CH_2_)_3_–CH_3_); 1.32 (2H, m, –N–CH_2_–CH_2_–CH_2_–CH_3_); 1.33 (2H, m, N–CH_2_–CH_2_–CH_2_ –CH_3_); 2.33 (3H, s, –CH_3_; 2.64 (2H, t, *J*_H-H_= 4.20 Hz N–CH_2_–CH_2_–CH_2_ –CH_3_)); 5.36 (1H, t, *J*_H-H_= 4.20 Hz, NH); 6.63 (1H, d, *J*_H-H_= 5.8 Hz, CH=CH-N); 7.35–7.56 (5H, m, H_arom_); 8.31 (1H, d, *J*_H-H_ = 5.8 Hz, CH=CH-N); ^13^C NMR(CDCl_3_): 13.71; 21.31; 32.62; 41.5; 88.3; 113.42; 117.01; 126.25–129.86; 136.88; 152.08; 155.34; 160.12. EIMS *m*/*z* (% relative abundance): 266 (M + H, 100), (19), HRMS (ESI-QTOF): Calcd for: C_17_H_20_N_3_M + H 266.1324; Found: 266.1123.

### 3.3. Antimicrobial Assay

- Microorganism Target and Growth Conditions

A panel of reference microorganisms was used to evaluate the antimicrobial profile of the synthesized novel 2-aminopyridine derivatives, namely(i)six Gram-positive bacteria(*Staphylococcus aureus* ATCC 25923, *Micrococcus luteus* ATCC 9341, *Listeria monocytogenes* ATCC 15313, *Bacillus cereus* ATCC 10876, *Bacillus subtilis* ATCC6633, and *Enterococcus faecalis* ATCC 49452), (ii) four Gram-negative strains (*Escherichia coli* ATCC 25912, *Pseudomonas aeruginosa* ATCC 27853, *Acinetobacter baumanii* ATCC19606, and *Salmonella typhimurium* ATCC 13311) and (iii) two yeasts (*Candida albicans* ATCC 10231 and *Candida albicans* ATCC 26790). The bacterial strains were cultured overnight at 37 °C in Brain-Heart Infusion Broth (BHIB, Biomedics, Spain),while *Candida albicans* strains in Sabouraud Dextrose Agar (SDA, Liofilchem, Abruzzi, Italy) for 48 h at 37.49 °C. Bacterial suspensions were adjusted to McFarland standard turbidity (0.5), which corresponds to 107–108 CFU·mL^−1^. However, the turbidity of yeast strains was 1 to 5 × 10^6^ CFU·mL^−1^.

- Disc-Diffusion Assay

The initial screening of the antimicrobial profile was performed using the disc-diffusion assay as was recommended by the **CLSI** [30]. Briefly, Petri dishes were seeded with adjusted inoculums strains using sterile swabs. Then, disc papers (06 mm in diameter) filled with 5 µg of each compound dissolved in DMSO were deposed on the Petri plates. All dishes were incubated at 37 °C for 24 h for bacteria and 48 h for yeast. Also, Gentamicin (10 µg/disc) and Amphotericin B (0.2 mg/disc) were tested as positive controls while DMSO represented a negative control. The diameters of the inhibition zones were then measured and expressed in millimeters. All the tests were performed as triplicates, means, and Standard Derivation (SD) were calculated using Past software (Version 3.22.).

- Minimum Inhibitory Concentration (MIC)

The Minimum Inhibitory Concentration (MIC) of the active compounds was determined by the serial dilution method in a 96-well plate according to the **CLSI** [30]. Hence, each well was inoculated with 100 µL of medium broth, and the initial concentration of active compounds was 20 mg·mL^−1^. After that, 100 µL of the initial concentration of active compounds was mixed with 100 µL of medium broth into the firstwell to obtain 1/2 dilution. Next, 100 μL of the first well were deposited in the second well to obtain 1/4. These dilutions were repeated until the last well (10th well). Then, 100 μL of the standardized suspension of sensitive microorganisms (1 × 10^6^ UFC·mL^−1^) were added to each well of the 96-well plate until reaching a final volume of 200 μL. A well (11th well) that contained medium with inoculums was considered a positive control, and the 12th well that contained just medium was considered a negative control. The plates were incubated for 24 h at 37 °C. The MIC was determined in the well of the lowest concentration, where no turbidity (i.e., no growth) was observed compared to chemical-free growth control. All tests were performed in a triplicate with results expressed in µg·mL^−1^.

### 3.4. Ligand and Target Preparations

The 3D structure of the 2-aminopyridine active compound (**2c**) was optimized using the semi-empirical method AM1 [31] implemented in Hyperchem 8.0.8 software [32]. The database was created by converting the compound into format*mdb. This database was used as the input for MOEdocking.

The 3D structures of proteins (Table 8) were downloaded from the https://www.rcsb.org website (5 February 2022). The proteins of *S.aureus* and *B. subtilis* were identified with their PDB codes. 4URM (resolution 2.94 Å) [33] and 2RHL (resolution 2.45 Å) [34] were respectively selected as antibacterial targets.

Both *S. aureus* (PDB ID: 4URM) and *B. subtilis* (PDB ID: 2RHL) X-ray crystals) were simplified by removing water molecules, ions, cofactors, and co-crystal ligands from their PDB structure. In addition, both *S. aureus* (2.94 Å) and *B. subtilis* (2.45 Å) targets possess a good resolution quality [35,36].

- Docking Protocol

Molecular docking is a computational technique used to find out the interaction of ligands within the active site of target proteins. Molecular Operating Environment (MOE) software [37] was used in molecular docking simulation. The same protocol steps were followed and employed in our previous investigations [38,39,40]. The following default parameters were used; Placement: Triangle Matcher, Rescoring 1: London dG.The London dG scoring function was employed to estimate the lowest score energy of the complex with the best pose of the tested compound.

- Protein-Ligand Pose and Affinity Predictions

Semi-flexible docking was performed to find compound **2c** affinity and interaction within the active site of the target proteins. The analysis of the results obtained during this work is based on the following five criteria:

(1) The low energy score of the complex indicates that the complex is stable;
(2) H-bond distances belonging to the interval between 2.5 and 3.1 Å are considered strong interactions and those ranging between 3.1 Å and 3.55 Å are assumed to be weak [18,19];
(3) The high number of interactions means that the stability of the complex is increased;
(4) The optimum range of the distance values of hydrophobic interactions is between 3.3–3.8 Å [41], although other researchers have suggested a relatively higher rate [42,43];
(5) The quality of the fit was evaluated based on the RMSD values of the compound according to the following ranges: rmsd (1.0 Å), good pose (1Å < rmsd ≤ 2.0 Å), close pose (2.0 Å < rmsd ≤ 3.0 Å), pose with errors (rmsd > 3.0 Å), and bad pose [44,45,46].

- ADME-T Prediction and Physicochemical properties

SwissADME server (http://www.swissadme.ch/) (5 February 2022) [47] was used to calculate the physicochemical properties in order to verify the different rules, namely Lipinski, Veber, and Ghose. In addition, we used the pkCSM server (http://biosig.unimelb.edu.au/pkcsm/prediction) (5 February 2022) [48] to predict the ADME-T (Absorption, Distribution, Metabolism, Excretion, and the Toxicity).

## 4. Conclusions

A series of 2-amino-3-cyanopyridines derivatives were synthesized by an efficient one-pot procedure based on the reaction of different enaminones with malononitrile and various primary amines under solvent-free conditions. It was found that this method was suitable for the rapid and clean synthesis of 2-aminopyridine derivatives. The antibacterial activities of some synthetic compounds were evaluated, and the results demonstrated that compound **2c** showed the highest activity against Gram-positive bacteria.

The results were also analyzed computationally using the molecular docking approach. From the docking analysis, it was also found that the tested compound **2c** exhibited the best antibacterial activity. The docking results correlate with the experimental results as the highly active compound (compound **2c**) showed good interactions with the active site residues of the target proteins compared to the native compounds. In addition, compound **2c** has improved pharmacokinetic properties, and it largely complies with the rules of Lipinski, Veber, and Ghose, as well as no toxicity, appeared inthis compound.

Thus, compound **2c** could be a strong candidate for more detailed studies to identify more effective exhibiting antimicrobial and antioxidant activities. Thus the further development of these compounds might be of great interest.

## Figures and Tables

**Table 1:** Synthesis of Enaminone **1a**–**c**.

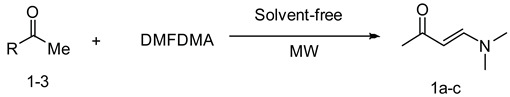			
Entry	R	Product	Yield (%)
1	Ph	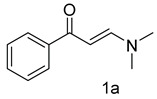	86
2	4-OCH_3_-C_6_H_4_-	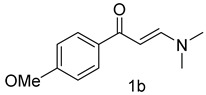	70
3	4-OCH_3_-C_6_H_4_-	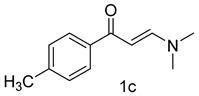	80

Reaction conditions: 1–3 (1 mmol), DMFDMA (1 mmol), Time: 5 min.

**Table 2:** Optimization of the reaction condition for **2b** synthesis.

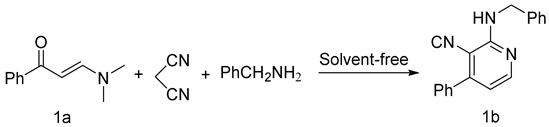			
Entry	Temperature (°C)	Time(h)	Yield (%)
1	25	24	0
2	40	24	20
3	60	6	49
4	80	3	75

Reaction conditions: 1a (1 mmol), malononitrile (1 mmol), and benzylamine (1 mmol).

**Table 3:** Multicomponent synthesis of 2-aminopyridines **2a**–**l**.

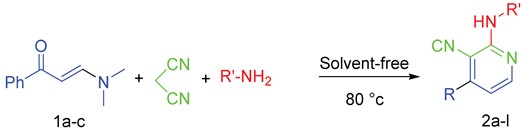			
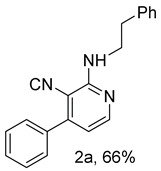	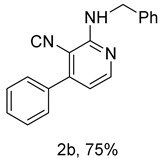	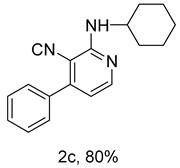	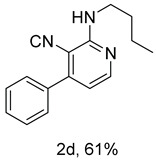
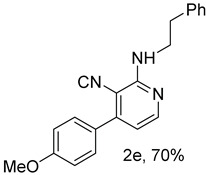	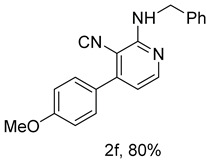	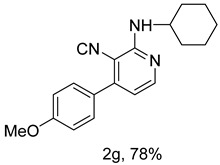	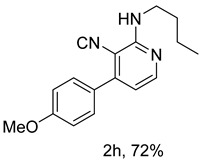
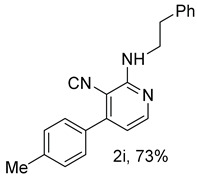	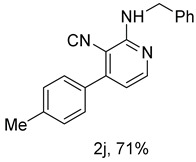	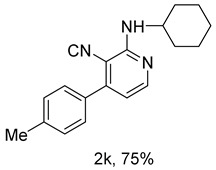	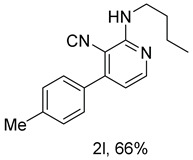

**Table 4:** Results of theantimicrobial assayusing the disk diffusion method.

Disc Charge 5 μg (Disc Diameter 6 mm)					Gentamycin 10 µg/Disk	Amph B 0.2 mg/Disk
Microorganisms	2a	2b	2c	2d		
*Bacillus cereus* ATCC 10876	-	-	11.33 ± 0.57	-	22	-
*Bacillus subtilis* ATCC6633	-	-	13 ± 0	-	20	-
*Enterococcus faecalis* ATCC49452	-	-	8.66 ± 0.57	-	13	-
*Staphylococcus aureus* ATCC 25923	-	-	9.66 ± 0.57	-	33	-
*Micrococcusluteus* ATCC 9341	-	-	9 ± 1	-		-
*Listeria monocytogenes* ATCC 15313	-	-	12 ± 1	-	12	-
*Acinetobacterbaumanii* ATCC19606	-	-	-	-	14	-
*Pseudomonas aeruginosa* ATCC27853	-	-	-	-	25	-
*Salmonella typhimurium* ATCC13311	-	-	-	-	26.5	-
*Escherichia coli* ATCC 25912	-	-	-	-	23	-
*Candida albicans* ATCC 10231	-	-	-	-	-	30 ± 0.0
*Candida albicans* ATCC 26790	-	-	-	-	-	32 ± 0.0

**Table 5:** MIC results of compound **2c**.

Microorganism Compounds	*B. cereus* ATCC 10876	*B. subtilis* ATCC6633	*M. luteus* ATCC 9341	*L. monocytogenes* ATCC 15313	*S. aureus* ATCC 25923	*E. faecalis* ATCC49452
2c MIC µg·mL^−1^	78 ± 0.000	39 ± 0.000	78 ± 0.000	156 ± 0.000	39 ± 0.000	78 ± 0.000
Gentamicine MIC µg·mL^−1^	0.625	5.2 ± 0.000	8 ± 0.000	2.21 ± 0.000	0,19 ± 0.000	0.78 ± 0.000

**Table 6:** S-score (Energy), RMSD, and interactions between compound **2c** and the active site residues of *S. aureus* (PDB ID: 4URM) and *B. subtilis* (PDB ID: 2RHL) targets.

*S. aureus* (PDB ID: 4URM)							
Compounds	Score (kcal/mol)	RMSD (Å)	Bonds between Atoms of Compounds and Active Site Residues				
			Atom of Compound	Involved Receptor Atoms	Involved Receptor Residues	Type of Interaction Bond	Distance (Å)
**2c**	−5.532	1.655	H	OE2	GLU(A:58)	Conventional H-bond	2.62
			O	HG1	THE(A:173)	ConventionalH-bond	2.88
			6-ring	HB2	ASN(A:54)	Pi–Sigma	2.38
			6-ring		ILE(A:86)	Pi–Alkyl	4.76
			C	C	PRO(A:87)	Alkyl	4.62
KBD	−6.383	2.408	H	OE1	GLU(A:58)	ConventionalH-bond	2.23
			H	OD2	ASP(A:81)	ConventionalH-bond	2.08
			H	OE1	GLN(A:91)	ConventionalH-bond	2.57
			5-ring	HB2	ASN(A:54)	Amide-Pi Stacked	2.61
			C	C	MET(A:94)	Alkyl	4.64
			C	C	ILE(A:86)	Alkyl	4.81
***B. Subtilis* (PDB ID: 2RHL)**							
**Compounds**	**Score (kcal/mol)**	**RMSD** **(Å)**	**Bonds between Atoms of Compounds and Active Site Residues**				
			**Atom of Compound**	**Involved Receptor** **Atoms**	**Involved Receptor** **Residues**	**Type of Interaction Bond**	**Distance** **(Å)**
**2c**	−6.389	1.706	N	H	GLY(A:110)	Conventional H-bond	2.38
			N	H	GLY(A:108)	ConventionalH-bond	3.10
			N	H	THE(A:109)	ConventionalH-bond	3.10
			C	6-ring	PHE(A:183)	Pi–Alkyl	5.18
			6-ring	NH1	ARG(A:143)	Pi–Cation	3.96
GDP	−7.843	1.160	O1B	H	GLY(A:110)	ConventionalH-bond	1.90
			O3B	H	GLY(A:108)	ConventionalH-bond	1.96
			H	OE2	GLU(A:139)	ConventionalH-bond	2.17
			O3	H	ARG(A:143)	ConventionalH-bond	2.05
			O6	HD21	ASN(A:25)	ConventionalH-bond	2.06
			6-ring	C	ALA(A:186)	Pi–Alkyl	5.04

**Table 7:** ADME-T and drug-likeness properties of compound 2c.

Entry	TPSA Å^2^		n-ROTB		MW g/moL	MLog P	n-ON Acceptors	n-OHNH Donors	Rules		
						WLog P			Lipinski	Veber	Ghose
Range	<140		<11		<500	≤5	<10	<5	≤1	≤1	≤1
**2c**	48.71		3		277.36	2.72	2	1	Accepted	Accepted	Accepted
						4.17					
**ADME-T**	**Absorption**		**Distribution**		**Metabolism**			**Excretion**		**Toxicity**	
	**Caco2** **(10^−6^ cm/s)**	**HIA** **%**	**CNS** **(log PS)**	**BBB** **(log BB)**	**CYP1A2 inhibitor**	**CYP2C19 inhibitor**	**CYP2D6 substrate**	**Renal OCT2** **substrate**	**Total** **Clearance** **(mL/min/kg)**	**AMES toxicity**	**hERG I/II** **Inhibitors**
**2c**	1.419	92.9	−1.837	0.22	No	Yes	No	Yes	0.81	No	No

ABS: Absorption, TPSA: Topological Polar Surface Area, n-ROTB: Number Of Rotatable Bonds, MW: Molecular Weight, M/WLog P: logarithm of partition coefficient of compound between n-octanol and water, n-ON acceptors: Number of hydrogen bond acceptors, n-OHNH donors: Number of hydrogen bonds donors. Caco-2: Colon adenocarcinoma, HIA: Human intestinal absorption, CNS: Central Nervous System permeability, BBB: Blood–Brain Barrier permeability.

**Table 8:** Some information related to both *S. aureus* (PDB ID: 4URM) and *B. subtilis* (PDB ID: 2RHL) targets.

Targets PDB	Methods	Microorganisms	Chain	Native Ligand
4URM	X-ray diffraction	*S. aureus*	A,B,C,D	XAM
2RHL	X-ray diffraction	*B. subtilis*	A,B	GDP

The native ligand of 4URM is XAM (Kibdelomycin), and 2RHL is GDP (guanosine-5′-diphosphate).

## Data Availability

Not applicable.
